# *Sophora flavescens* Aiton Decreases MPP^+^-Induced Mitochondrial Dysfunction in SH-SY5Y Cells

**DOI:** 10.3389/fnagi.2018.00119

**Published:** 2018-04-24

**Authors:** Hee-Young Kim, Hyongjun Jeon, Hyungwoo Kim, Sungtae Koo, Seungtae Kim

**Affiliations:** ^1^Korean Medicine Research Center for Healthy Aging, Pusan National University, Yangsan, South Korea; ^2^Department of Korean Medical Science, School of Korean Medicine, Pusan National University, Yangsan, South Korea; ^3^Division of Pharmacology, School of Korean Medicine, Pusan National University, Yangsan, South Korea

**Keywords:** Parkinson’s disease, *Sophora flavescens* Aiton, SH-SY5Y, MPP^+^, mitochondrial dysfunction

## Abstract

*Sophora flavescens* Aiton (SF) has been used to treat various diseases including fever and inflammation in China, South Korea and Japan. Several recent reports have shown that SF has anti-inflammatory and anti-apoptotic effects, indicating that it is a promising candidate for treatment of Parkinson’s disease (PD). We evaluated the protective effect of SF against neurotoxin 1-methyl-4-phenylpyridinium ion (MPP^+^)-induced mitochondrial dysfunction in SH-SY5Y human neuroblastoma cells, an *in vitro* PD model. SH-SY5Y cells were incubated with SF for 24 h, after which they were treated with MPP^+^. MPP^+^-induced cytotoxicity and apoptosis were confirmed by 3-(4,5-dimethyl-thiazol-2-yl)-2,5-diphenyl tetrazolium bromide assay and terminal deoxynucleotidyl transferase-mediated biotinylated UTP nick end labeling assay. MitoSOX red mitochondrial superoxide indicator, tetramethylrhodamine methyl ester perchlorate and Parkin, PTEN-induced putative kinase 1 (PINK1), and DJ-1 immunofluorescent staining were conducted to confirm the mitochondrial function. In addition, western blot was performed to evaluate apoptosis factors (Bcl-2, Bax, caspase-3 and cytochrome c) and mitochondrial function-related factors (Parkin, PINK1 and DJ-1). SF suppressed MPP^+^-induced cytotoxicity, apoptosis and collapse of mitochondrial membrane potential by inhibiting the increase of reactive oxidative species (ROS) and DNA fragmentation, and controlling Bcl-2, Bax, caspase-3 and cytochrome *c* expression. Moreover, it attenuated Parkin, PINK1 and DJ-1 expression from MPP^+^-induced decrease. SF effectively suppressed MPP^+^-induced cytotoxicity, apoptosis and mitochondrial dysfunction by regulating generation of ROS, disruption of mitochondrial membrane potential, mitochondria-dependent apoptosis and loss or mutation of mitochondria-related PD markers including Parkin, PINK1 and DJ-1.

## Introduction

Parkinson’s disease (PD) is an incurable neurodegenerative disorder characterized by bradykinesia, muscle rigidity, tremor and postural instability (Olanow and Tatton, 1999). These features of PD are associated with the appearance of Lewy bodies in the neuronal cytoplasm and progressive degeneration and death of dopaminergic neurons (DA) in the substantia nigra pars compacta (Moore et al., 2005). Although the exact etiology of PD has yet to be elucidated, many recent studies have reported that oxidative stress and mitochondrial dysfunction are involved in progression of PD (Requejo-Aguilar and Bolaños, 2016). A neurotoxin, 1-methyl-4-phenyl-1,2,3,6-tetrahydropyridine (MPTP), is widely used for PD research because it selectively destroys DA in animals (Fernández-Moriano et al., 2015). MPTP is metabolized into 1-methyl-4-phenylpyridinium ion (MPP^+^) by monoamine oxidase B in the brain, and MPP^+^ disturbs mitochondrial respiration by inhibiting mitochondrial complex I. This process leads to abnormal mitochondrial metabolism and generation of reactive oxygen species (ROS) like pathogenesis of PD (Cleeter et al., 1992). In addition, increased cellular ROS production has been implicated in neurodegenerative diseases such as PD and Alzheimer’s disease (Fernández-Moriano et al., 2015). According to several recent studies, MPP^+^ causes opening of mitochondrial permeability transition pores, mitochondrial membrane potential disruption, impairment of ATP production and ROS generation, which induces apoptosis of DA (Lee et al., 2011; Requejo-Aguilar and Bolaños, 2016). Several PD-related substances, such as α-Synuclein, Parkin, PTEN-induced putative kinase 1 (PINK1), DJ-1 and Leucine-rich repeat kinase 2 (LRRK2), are involved in mitochondrial function in DA; therefore, loss or mutation of these substances causes cell death (Um et al., 2009; Fernández-Moriano et al., 2015; Requejo-Aguilar and Bolaños, 2016).

Currently, prospective drugs for PD do not exist. Several effective drugs, such as levodopa, can enhance clinical symptoms, but these drugs not only suppress progression of PD, but also induce side effects (Hu et al., 2011). Therefore, alternative medicine, curative foods, and phytochemicals are considered to have the potential for prevention or treatment of PD. Recently, beneficial effects of herbal medicines (Chen et al., 2007; Kim et al., 2009), ginseng (Kim et al., 2016), astaxanthin (Lee et al., 2011), and lycopene (Yi et al., 2013) in PD-like experimental models have been demonstrated.

*Sophora flavescens* Aiton (SF), a kind of deciduous shrub, is widespread in East Asia (He et al., 2015). Diverse flavonoids, alkaloids, and terpenoids have been found in SF, with flavonoids (e.g., kushenol, kurarinone and sophoflavescenol) and alkaloids (e.g., matrine and oxymatrine) comprising the major active components of SF (He et al., 2015). SF has been used for treatment of fever, pain, inflammation, ulcer, numbness and several skin diseases as traditional medicine in China, South Korea and Japan (Kim et al., 2012; He et al., 2015). Moreover, SF or its compounds have been shown to have anti-oxidative (Piao et al., 2006), anti-cancer (Sun et al., 2007; Liu et al., 2010), anti-arthritic (Jin et al., 2010), anti-ulcerative (Yamahara et al., 1990) and anti-dermatitis (Kim et al., 2012) effects, and recent studies have shown that it has neuroprotective effects. Extract of SF suppressed sodium nitroprusside-induced apoptosis in SH-SY5Y cells, while focal cerebral ischemia induced neuronal death in rats (Park et al., 2009) and enhanced axonal growth in mice (Tanabe et al., 2015), and oxymatrine extracted from SF prevented neuronal cell death in rat brains subjected to cerebral ischemia-reperfusion damage (Li et al., 2011). However, it is still not known if SF can also protect DA. Therefore, in the present study, we examined the protective effects of SF against MPP^+^-induced cytotoxicity and mitochondrial dysfunction in human neuroblastoma SH-SY5Y cells.

## Materials and Methods

### Preparation of SF Extract

SF was purchased from Kwangmyungdang Medical Herbs (Ulsan, South Korea). For extraction, 50 mg SF was immersed in absolute methanol (1000 mL) and sonicated for 30 min, extracted for 24 h, filtered through filter paper, and dried in a vacuum evaporator (Eyela, Japan). Finally, the extract was lyophilized by freeze-drying (Labconco, Kansas City, MO, USA). The yield value of powdered SF was 12.9%. The methanol extract of SF has been deposited at the Division of Pharmacology, School of Korean Medicine, Pusan National University (Voucher No. MH2010-004). The dark green colored SF extract was used to dissolve in phosphate-buffered saline (PBS) in all of experiments.

### Cell Culture

SH-SY5Y cells, a human neuroblastoma cell line (KCLB, Korean cell line bank, Seoul, South Korea), were cultured in Dulbecco’s Modified Eagle’s Medium (Welgene, Daegu, South Korea) supplemented with 10% (v/v) heat-inactivated fetal bovine serum (Welgene) and 100 units/mL penicillin/streptomycin (Welgene). Cells were maintained at 37°C in a humidified 95% air and 5% CO_2_ atmosphere.

### Cell Viability Assay

Cell viability was determined by 3-(4,5-dimethyl-thiazol-2-yl)-2,5-diphenyl tetrazolium bromide (MTT; Duchefa Biochemie, Haarlem, Netherlands) assay. Cells were pre-incubated in 96-well plates at a confluence of 1 × 10^5^ cells/mL, then treated with SF for 24 h, after which they were treated with MPP^+^ for 24 h. Then, they were grown in 0.5 mg/mL MTT for 4 h. After medium was aspirated, 100 μL of dimethyl sulfoxide was added to dissolve the purple formazan crystals. Following incubation for 30 min, plates were read at 540 nm.

### TUNEL Assay

Terminal deoxynucleotidyl transferase-mediated biotinylated UTP nick end labeling (TUNEL) assay was performed using a fluorometric TUNEL assay kit (Promega, Madison, WI, USA). Cells were grown in 8-well chamber slides (SPL Life Sciences, Pocheon, Korea) to a confluence of 3 × 10^4^ cells/mL. Next, cells were incubated with or without SF for 24 h, then incubated with or without MPP^+^ for 24 h. The, medium was then aspirated and fixed with 4% (v/v) paraformaldehyde for 30 min at 4°C, after which cells were stained by TUNEL and DAPI according to the manufacturer’s protocols. Finally, fluorescence was detected by LSM 700 confocal laser scanning microscope (Zeiss, Oberkochen, Germany).

### Western Blot

Cells were cultured in 6-well plates to a confluence of 1 × 10^5^ cells/mL. Next, the cells were incubated with or without SF for 24 h, re-incubated with or without MPP^+^ for 24 h, then detached with ice cold RIPA buffer (Armnesco, Solon, OH, USA) and centrifuged at 13,000 rpm for 15 min at 4°C. The protein concentration was subsequently determined using a BioRad protein assay kit (Hercules, CA, USA). Protein samples (20 μg) were then separated by sodium dodecyl sulfate–polyacrylamide gel electrophoresis and electrotransferred onto a 0.45 μm nitrocellulose blotting membrane (GE Healthcare UK Ltd, Little Chalfont, UK). The blots were incubated with anti-Bcl-2 (diluted 1:200), anti-Bax (1:1000), anti-cytochrome c (1:200), anti-cleaved caspase-3 (1:1000), anti-Parkin (1:1000), anti-PINK1 (1:200), anti-DJ-1 (1:1000) or anti-β-actin (1:200) primary antibodies while immersed in 5% skim milk or 5% Bovine serum albumin overnight at 4°C, after which they were incubated with secondary antibodies for 1 h at room temperature. The membranes were then washed several times with PBS containing 0.05% (v/v) Tween 20, after which they were visualized with ECL reagent (Thermo Fisher Scientific Inc., Rochford, IL, USA). All primary antibodies were purchased from Santa Cruz Biotechnology (Santa Cruz, CA, USA) except anti-cleaved caspase-3 and anti-DJ-1 (Cell Signaling Technology, Inc., Beverly, MA, USA).

### Measurement of Mitochondrial Membrane Potential and Mitochondrial Reactive Oxygen Species

Cells were cultured in eight well-chamber slides with or without SF for 24 h, then re-incubated with or without MPP^+^ for 24 h, after which 250 nM tetramethylrhodamine methyl ester perchlorate (TMRM; Invitrogen, Carlsbad, CA, USA) or 5 μM MitoSOX red mitochondrial superoxide indicator (MitoSOX; Invitrogen) was treated and the samples were incubated for 30 min or 10 min, respectively at 37°C. Cells were washed three times with PBS, after which they were counterstained with mitotracker green (Invitrogen, Carlsbad, CA, USA). Following counterstaining, the cells were fixed with 4% (v/v) paraformaldehyde (25 min, 4°C), and mounted with Vectashield mounting medium with DAPI (Vector, Burlingame, CA, USA). Finally, images of the slides were captured using a LSM 700 confocal laser scanning microscope (Zeiss).

### Immunofluorescent Staining

Cells cultured in eight well-chamber slides were fixed with 4% (v/v) paraformaldehyde in PBS for 20 min, then permeabilized with 0.1% (v/v) Triton X-100 in PBS for 15 min at room temperature. Next, the cells blocked with 10% normal goat serum (NGS) in PBS for 1 h at room temperature. The cells were subsequently incubated with primary antibodies (anti-Parkin, anti-PINK1 and anti-DJ-1) dissolved in 10% NGS at 4°C, overnight, after which they were incubated with fluorophore-conjugated secondary antibodies anti-rabbit Alexa-488 IgG or anti-mouse Alexa-594 IgG (Molecular Probes, Eugene, OR, USA) for 1 h at room temperature in the dark. The cells were rinsed with 1% NGS and mounted with Vectashield mounting medium with DAPI (Vector), after which images of the cells were captured using a LSM 700 confocal laser scanning microscope (Zeiss). The numbers of Parkin, PINK1, or DJ-1-positive cells were counted on each capture. All cell counts were confirmed three times.

### Statistical Analysis

All data are drawn from triplicate experiments, and presented as the means ± SD. Differences between the mean values were assessed by independent *t*-test in MTT assay, and one-way analysis of variance (ANOVA) with Duncan’s multiple-range tests in other experiments. All statistical analyses were performed using IBM SPSS Statistics Ver. 22 (IBM Co., Armonk, NY, USA). Differences with a *p* < 0.05, was considered statistically significant.

## Results

### SF Protects SH-SY5Y Cells Against MPP^+^-Induced Cytotoxicity and Apoptosis

**Figure 1:**
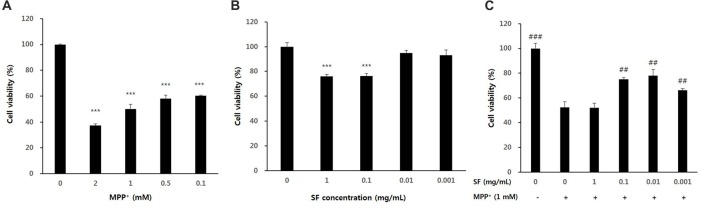
Cell viability after 1-methyl-4-phenylpyridinium ion (MPP^+^) or *Sophora flavescens* Aiton (SF) treatment of SH-SY5Y cells. Dose-dependent effects were observed in SH-SY5Y cells treated with MPP^+^ **(A)**, SF **(B)**, or both SH-SY5Y and MPP^+^ **(C)**. Values are the means ± SD (*n* = 6). In **(A,B)**, ***p < 0.001, compared with control (untreated group). In **(C)**, ^##^*p* < 0.01 and ^###^*p* < 0.001, compared with the group treated by MPP^+^ alone.

SH-SY5Y cells were treated with various concentrations of SF and MPP^+^ to confirm their cytotoxicity. When the cells were exposed to MPP^+^ for 24 h, cell viability was significantly decreased in a dose-dependent manner. When compared with the control cells, cells exposed to 1 mM MPP^+^ for 24 h showed 50.1 ± 3.4% of the cell viability. The dose of 1 mM MPP^+^ was also used in previous studies (Lee et al., 2011; Janhom and Dharmasaroja, 2015); therefore, a dose of 1 mM MPP^+^ was used for further experiments (Figure 1A). SH-SY5Y cells treated with 0.001, 0.01, 0.1 or 1 mg/mL of SF showed 93.0 ± 4.5, 94.8 ± 2.3%, 76.3 ± 2.0, and 76.0 ± 1.6% cell viability relative to the control, respectively (Figure 1B). Pretreatment of SH-SY5Y cells with SF at 0.001, 0.01, or 0.1 mg/mL for 24 h followed by 1 mM MPP^+^ for 24 h significantly attenuated MPP^+^-evoked toxicity (*p* < 0.01), with 0.01 mg/mL SF pretreatment resulting in the highest cell viability (77.9 ± 5.0%; Figure 1C). Therefore, a dose of 0.01 mg/mL SF was used further experiments.

**Figure 2:**
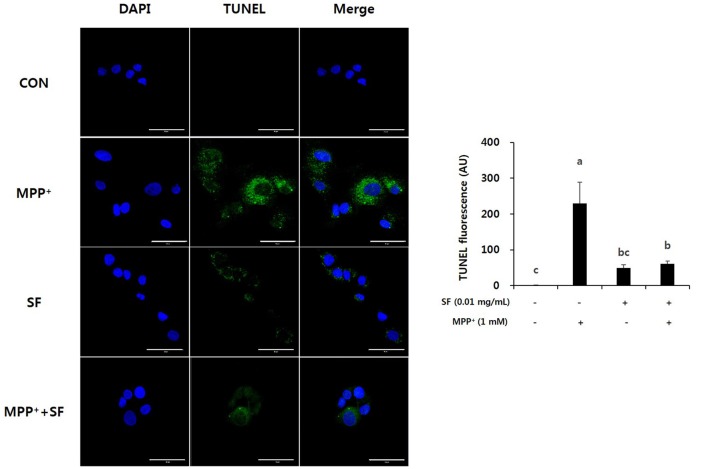
SF attenuated MPP^+^-induced apoptosis in SH-SY5Y cells. Cells were treated with 0.01 mg/mL SF for 24 h, then exposed to 1 mM MPP^+^ for 24 h and stained with DAPI and TUNEL. TUNEL staining showed that MPP^+^ increased apoptotic DNA fragmentation, but SF suppressed it. Scale bar = 50 μm. Values are the means ± SD (three slides per group). ^a–c^Means with different letters are significantly different (*p* < 0.05) by Duncan’s multiple-range test.

To confirm the protective effects of SF on MPP^+^-induced apoptosis, a TUNEL assay was performed on SH-SY5Y cells. As shown in Figure 2, cells incubated with MPP^+^ alone showed the highest level of TUNEL positive fluorescence. However, cells treated with SF (presence or absence of MPP^+^) showed significantly lower apoptotic fluorescence than those treated with MPP^+^ alone. These results indicate that SF reduced MPP^+^-induced apoptotic DNA fragmentation in SH-SY5Y cells.

### SF Suppresses MPP^+^-Mediated Intracellular ROS Generation and Disruption of Mitochondrial Membrane Potential

**Figure 3:**
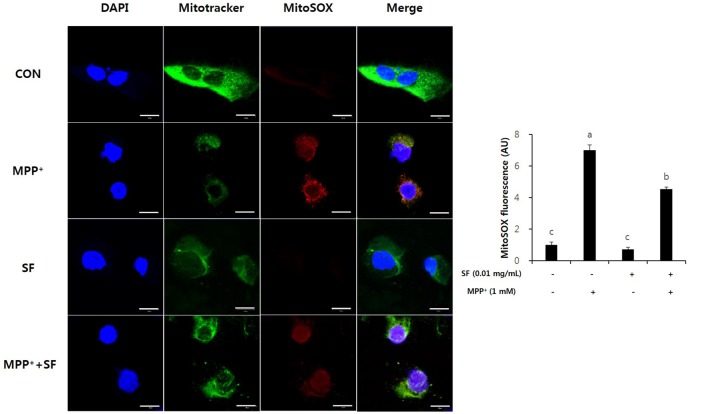
SF attenuated MPP^+^-mediated reactive oxidative species (ROS) generation in SH-SY5Y cells. Cells were exposed to 1 mM MPP^+^ for 24 h in the absence or presence of 0.01 mg/mL SF for 24 h, then stained with DAPI, MitoSOX and Mitotracker. MPP^+^ increased mitochondria-derived ROS generation, which was suppressed by SF. Scale bar = 50 μm. Values are the means ± SD (three slides per group). ^a–c^Means with different letters are significantly different (*p* < 0.05) by Duncan’s multiple-range test.

MitoSOX selectively targets the mitochondria and exhibits red fluorescence in the mitochondria of live cells after oxidation by superoxide. MPP^+^-treated SY-SH5Y cells showed significant increases of MitoSOX red fluorescence emission, indicating an increase of superoxide in mitochondria. However, the SF and MPP^+^-treated cells showed lower red fluorescence emission than cells treated with only MPP^+^. In addition, the intensity of the red signal in SF-only treated cells was similar to that in control cells. These results suggest that SF diminished MPP^+^-induced ROS generation in mitochondria (Figure 3).

**Figure 4:**
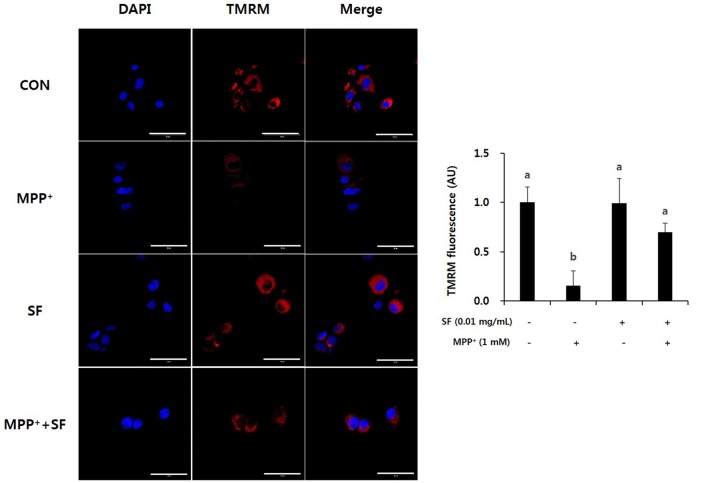
SF attenuated MPP^+^-mediated collapse of mitochondrial membrane permeability in SH-SY5Y cells. Cells were exposed to 1 mM MPP^+^ for 24 h in the absence or presence of 0.01 mg/mL SF for 24 h, stained with tetramethylrhodamine methyl ester perchlorate (TMRM), then visualized by fluorescence microscopy and the intensity of fluorescence was calculated. MPP^+^ collapsed mitochondrial membrane permeability, which was prevented by SF. Scale bar = 50 μm. Values are the means ± SD (three slides per group). ^a,b^Means with different letters are significantly different (*p* < 0.05) by Duncan’s multiple-range test.

The cell-permeant dye TMRM is accumulated in active mitochondria with intact membrane potentials; thus, bright red fluorescence exists in the healthy mitochondria of live cells. Induction of MPP^+^ causes abnormal mitochondrial membrane potential, so cells treated with MPP^+^ alone showed lower levels of TMRM positive signal than control cells. However, the SF and MPP^+^-treated cells showed significantly stronger signals than cells treated only with MPP^+^ (Figure 4). These findings indicate that SF attenuated MPP^+^-induced mitochondrial membrane depolarization. The signal in cells treated with SF alone did not differ significantly from the control cells.

### SF Regulates Protein Expression of Bcl-2 and Bax

**Figure 5:**
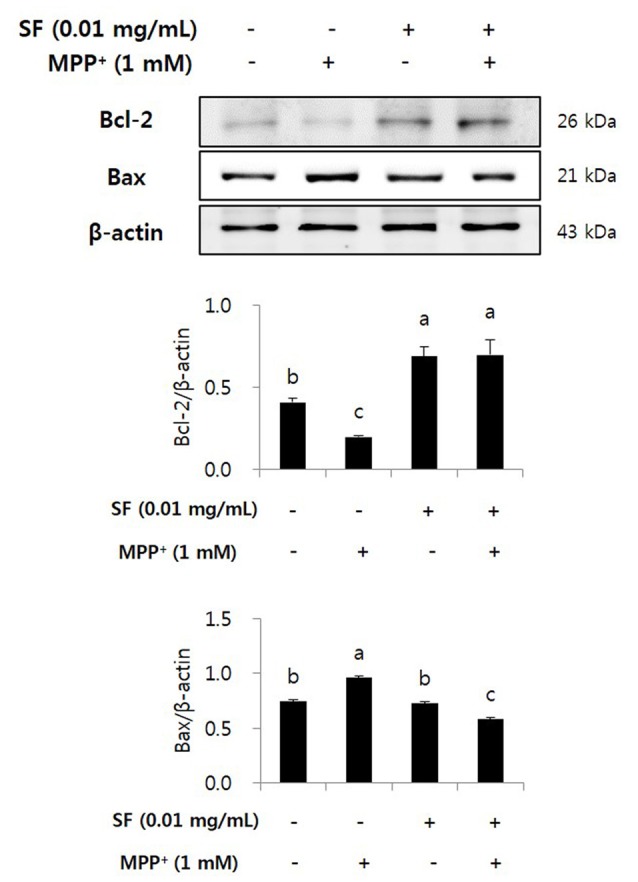
SF regulated MPP^+^-induced changes in Bcl-2 and Bax expression in SH-SY5Y cells. Cells were exposed to 1 mM MPP^+^ for 24 h in the absence or presence of 0.01 mg/mL SF for 24 h, then harvested and subjected to Western blotting using the antibodies against Bcl-2 and Bax. SF alleviated MPP^+^-induced down-regulation of Bcl-2 and up-regulation of Bax. Values are the means ± SD (*n* = 3). ^a–c^Means with different letters are significantly different (*p* < 0.05) by Duncan’s multiple-range test.

The expression of anti- and pro-apoptotic proteins Bcl-2 and Bax on SF and/or MPP^+^ treated SH-SY5Y cells was confirmed by western blotting. The expression of anti-apoptotic Bcl-2 decreased significantly in response to MPP^+^ treatment; however, SF pretreatment prevented the decrease significantly. Moreover, MPP^+^ led to up-regulation of Bax expression, which was significantly suppressed by SF pretreatment (Figure 5).

### SF Inhibits MPP^+^-Mediated Activation of Cytochrome c and Caspase-3

**Figure 6:**
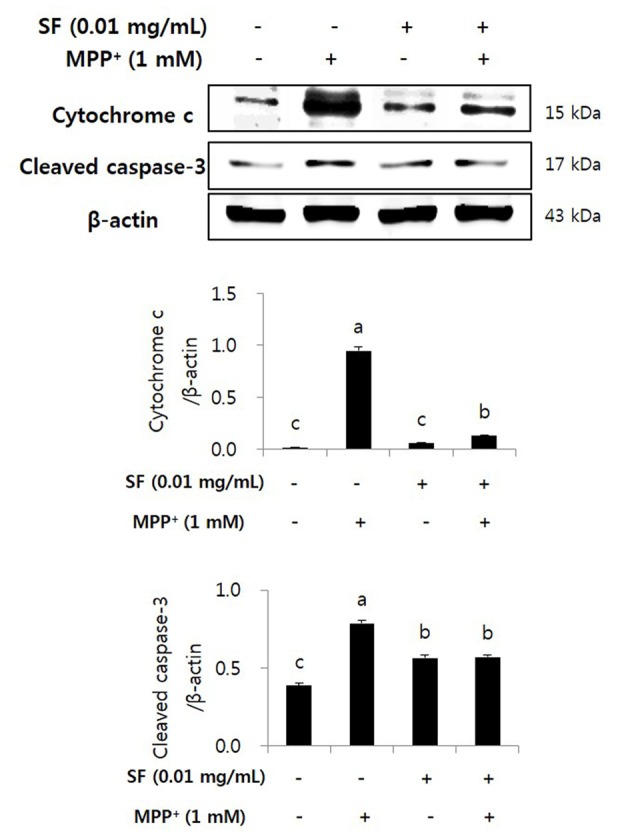
SF inhibited MPP^+^-induced release of cytochrome c and activation of caspase-3 in SH-SY5Y cells. Cells were exposed to 1 mM MPP^+^ for 24 h in the absence or presence of 0.01 mg/mL SF for 24 h. Cells were harvested and subjected to Western blotting using antibodies against cytochrome c and cleaved caspase-3. SF suppressed MPP^+^-induced release of cytochrome c and activation of caspase-3. Values are the means ± SD (*n* = 3). ^a–c^Means with different letters are significantly different (*p* < 0.05) by Duncan’s multiple-range test.

Cytochrome c release and caspase cascades play major roles in apoptosis. Cytochrome c released from mitochondria stimulates caspase-9 and caspase-3, which causes apoptosis of cells (Fernández-Moriano et al., 2015). MPP^+^-treated cells showed significantly higher level of cytochrome c than the other groups, however, the cells treated with MPP^+^ and SF showed significantly lower level than the MPP^+^ alone. MPP^+^ increased cleaved caspase-3 expression; however, SF significantly attenuated it (*p* < 0.05) and the level was similar to that of the SF alone (Figure 6).

### SF Suppresses MPP^+^-Mediated Reduction of Parkin, PINK1 and DJ-1

**Figure 7:**
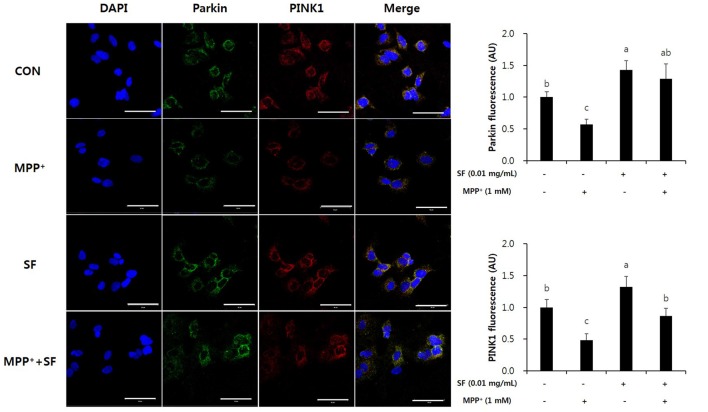
SF suppressed MPP^+^-mediated loss of Parkin and PTEN-induced putative kinase 1 (PINK1) in SH-SY5Y cells. Cells were exposed to 1 mM MPP^+^ for 24 h in the absence or presence of 0.01 mg/mL SF for 24 h, after which they were subjected to immunofluorescent staining using antibodies against Parkin and PINK1. MPP^+^ suppressed Parkin and PINK1 expression, while SF enhanced them. Scale bar = 50 μm. Values are the means ± SD (three slides per group). ^a–c^Means with different letters are significantly different (*p* < 0.05) by Duncan’s multiple-range test.

**Figure 8:**
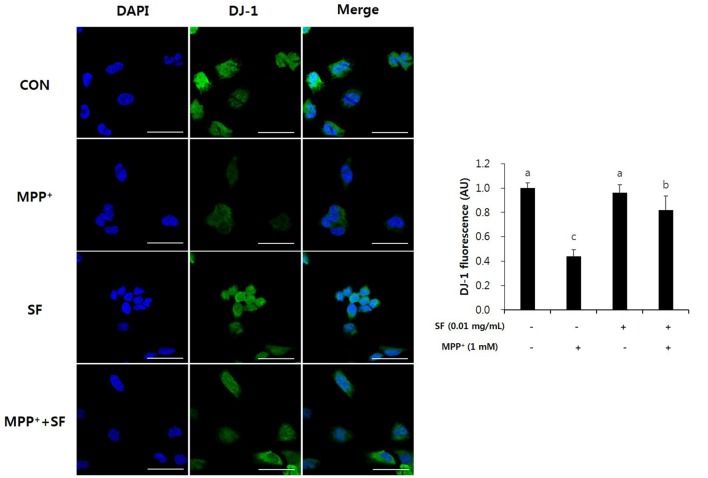
SF attenuated MPP^+^-mediated loss of DJ-1 in SH-SY5Y cells. Cells were exposed to 1 mM MPP^+^ for 24 h in the absence or presence of 0.01 mg/mL SF for 24 h, then subjected to immunofluorescent staining using antibody against DJ-1. SF enhanced the MPP^+^-induced decrease of DJ-1. Scale bar = 50 μm. Values are the means ± SD (three slides per group). ^a–c^Means with different letters are significantly different (*p* < 0.05) by Duncan’s multiple-range test.

Parkin, PINK1 and DJ-1 are involved in mitochondrial function and play important roles in the pathophysiology of PD. Immunofluorescent staining revealed that the levels of Parkin and PINK1 in SF-treated cells with no MPP^+^ were higher than those in control cells. Moreover, MPP^+^ treatment reduced the levels of Parkin and PINK1 compared with no MPP^+^ treatment, but SF pretreatment attenuated the MPP^+^-induced reduction significantly (Figure 7). The level of DJ-1 was also decreased in MPP^+^-treated cells compared control cells; however, SF prevented the decrease (Figure 8).

**Figure 9:**
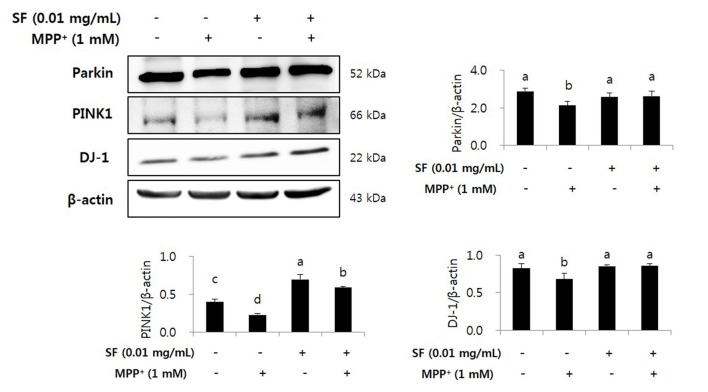
SF inhibited loss of Parkinson’s disease (PD) related markers Parkin, PINK1 and DJ-1 in MPP^+^-induced SH-SY5Y cells. Cells were exposed to 1 mM MPP^+^ for 24 h in the absence or presence of 0.01 mg/mL SF for 24 h, then harvested and subjected to Western blotting using antibodies against Parkin, PINK1 and DJ-1 with whole cell lysate. MPP^+^ suppressed Parkin, PINK1 and DJ-1 expression, while SF enhanced them. Values are the means ± SD (*n* = 3). ^a–d^Means with different letters are significantly different (*p* < 0.05) by Duncan’s multiple-range test.

We confirmed the levels of Parkin, PINK1, and DJ-1 by Western blotting. MPP^+^ treatment reduced the levels of Parkin, PINK1, and DJ-1 in SH-SY5Y cells; however, SF pretreatment significantly attenuated these decreases. Both the SF-treated and the SF and MPP^+^-treated cells showed comparable protein expression of Parkin and DJ-1 compared to the control cells and higher expression of PINK1 compared to the control and the MPP^+^-only treated cells (Figure 9).

## Discussion

In the present study, we demonstrated neuroprotective effect of SF in an *in vitro* PD model using neurotoxin MPP^+^ and SH-SY5Y human neuroblastoma cells. The SH-SY5Y human neuroblastoma cell line is widely used for PD research because the cells have many characteristics of human DA (Sheehan et al., 1997). MPP^+^, a metabolite of MPTP, has been used for PD research because it induces PD-like symptoms and degeneration of DA *in vitro* and *in vivo* (Langston et al., 1984). MPP^+^-induced cell death is related to several factors such as oxidative stress (Jung et al., 2007), opening of mitochondrial permeability transition pores, and disruption of the mitochondrial membrane potential make sound it better in English.(Seaton et al., 1997). These abnormal mitochondrial conditions can cause production of ROS, impairment of energy production and cell death (Saporito et al., 2000; Alcaraz-Zubeldia et al., 2001).

We confirmed the protective effects of SF against MPP^+^-induced neurotoxicity and apoptosis in SH-SY5Y cells by MTT and TUNEL assay. MPP^+^ is known as a ROS generative neurotoxin (Johannessen et al., 1986). ROS generated by MPP^+^ are involved in DNA and mitochondrial damage, as well as apoptotic cell death (Cassarino et al., 1999). In this study, MPP^+^ induced cell death as previously reported. In addition, we found that SF pretreatment significantly attenuated DNA fragmentation and apoptosis (Figures 1, 2), suggesting that SF can prevent MPP^+^-induced apoptosis in SH-SY5Y cells. And partially SF treated cells showed a little unhealthy during the TUNEL assay (Figure 2). SF contains alkaloids including matrine and oxymatrine, the two alkaloids has been demonstrated to possess strong anti-tumor activities (Liu et al., 2014). SH-SY5Y is a human derived cell line and a kind of neuroblastoma, therefore the unhealthy cells treated with SF might be due to the anti-tumor activity of matrine and oxymatrine. However the influence of them was limited because 0.01 mg/mL SF showed 94.8% of cell viability and it is not significantly different level comparing to control cells (Figure 1B).

Oxidative stress is a major factor inducing apoptosis in cells (Zhang et al., 2000) and associated with neuronal damage and behavioral impairment in PD (Venkatesh Gobi et al., 2018). Mitochondria are vulnerable to oxidative stress; thus, excessive production of ROS leads to abnormal mitochondrial morphology, opening of the mitochondrial transition pore, and collapse of mitochondrial membrane potential (Cassarino et al., 1999; Büeler, 2009; Yao and Wood, 2009). These changes in mitochondrial membrane cause critical event in the process leading to apoptosis (Dispersyn et al., 1999). The oxidative stress-mediated apoptosis is related to the activation of Bcl-2 family proteins, release of cytochrome c, and activation of caspase cascades (O’Malley et al., 2003; Ahn et al., 2009). Bcl-2 and Bax are members of the Bcl-2 family that serve as important apoptosis-related factors. Anti-apoptotic Bcl-2 and pro-apoptotic Bax regulate the membrane permeability of mitochondria as well as discharge of cytochrome c from mitochondria to cytosol (Crompton, 2000), especially Bcl-2 stabilizes inner mitochondrial transmembrane (Dispersyn et al., 1999) and prevents the release of cytochrome c (Yang et al., 1997). Caspase-3 is an important factor in downstream mitochondrial dysfunction related cell apoptosis (Strasser et al., 2000) that is activated by several apoptotic pathways, including the cytochrome c-dependent pathway. In the present study, MPP^+^ treatment accumulated ROS in the mitochondria of SH-SY5Y cells, but SF pretreatment significantly prevented this (*p* < 0.05; Figure 3). Moreover, SF up-regulated Bcl-2 and down-regulated Bax in cells (Figure 5) and SF inhibited MPP^+^-induced activation of cytochrome c and caspase-3 (Figure 6), which suggests that SF effectively suppressed mitochondria-dependent apoptosis in MPP^+^-treated SH-SY5Y cells. In addition, mitochondrial membrane potential levels in the SF-treated cells were similar to those in control cells, regardless of MPP^+^ treatment (Figure 4), indicating that SF effectively suppressed ROS generation and disruption of mitochondrial membrane potential.

Several genetic problems are involved in PD, especially mutations of Parkin, PINK1, and DJ-1, which are related to autosomal-recessive PD (Kitada et al., 1998; Bonifati et al., 2003; Valente et al., 2004). These genes play roles in neuronal cell apoptosis and mitochondrial functions such as ROS production, changes in mitochondrial morphology, mitochondrial respiration, and mitochondrial transportation (Requejo-Aguilar and Bolaños, 2016). In addition, Parkin, PINK1, and DJ-1 are involved in up-regulation of ATP production, mitochondrial membrane potential and mitophagy, as well as down-regulation of the mitochondrial permeability transition pore (Requejo-Aguilar and Bolaños, 2016). Parkin prevents DA death by suppression of α-synuclein aggregation (Bian et al., 2012). When mitochondrial membrane potential collapses, PINK1 recruits Parkin, and Parkin-mediated mitophagy is triggered to maintain a normal condition of mitochondria (Geisler et al., 2010). Ca^2+^ transfer is important process for mitochondrial respiration in neuronal cells, and Parkin, PINK1 and DJ-1 are involved in Ca^2+^ homeostasis by controlling connection of the endoplasmic reticulum-mitochondria and efflux of Ca^2+^ in the mitochondria (Calì et al., 2013a,b). Loss of DJ-1 causes mitochondrial fragmentation and increases sensibility against toxins including MPTP and paraquat; however, high levels of DJ-1 expression protect the cells from the toxins (Kim et al., 2005; Yang et al., 2007; Requejo-Aguilar and Bolaños, 2016). In the present study, MPP^+^ treatment significantly reduced the levels of Parkin, PINK1, and DJ-1 expression; however, SF attenuated these reductions. Moreover, SF treatment with no MPP^+^ increased the levels of Parkin and PINK1 significantly (Figures 7–9). These results suggest that SF not only protects cells against MPP^+^ toxicity, but also recovers Parkin, PINK1 and DJ-1 expression from MPP^+^-induced downregulation.

Regulation of intracellular ROS and normalization of mitochondrial function can control neurodegenerative events, especially apoptosis, in PD. In the present study, SF significantly suppressed MPP^+^-induced ROS generation, collapse of mitochondrial membrane potential, mitochondria-dependent apoptosis and mitochondria-related factors in SH-SY5Y cells, indicating that the protective effects of SF are due to alleviation of MPP^+^-induced mitochondrial dysfunctions. However, it should be noted that there were several limitations that should be addressed in future studies. First, the active compound in SF was not investigated in this study. Second, SH-SY5Y is neuroblastoma cell line, so additional *ex vivo* or *in vivo* study is necessary to confirm that SF actually alleviates PD symptoms and that the protective mechanism works in normal neurons.

## Conclusion

The results of the present study showed that SF has neuroprotective effects against MPP^+^-induced cell death in SH-SY5Y cells. Specifically, SF suppressed MPP^+^-mediated ROS generation and collapse of mitochondrial membrane potential. Moreover, SF controlled apoptosis-related protein expression of Bcl-2 and Bax, caspase-3, release of cytochrome c and PD-related factors including Parkin, PINK1 and DJ-1 in SH-SY5Y cells. These results suggest that SF has the potential for use in neuroprotective therapeutics of PD.

## Author Contributions

H-YK performed MTT assay, the acquisition of Western blotting and immunofluorescent data, conducted statistical analysis and drafted the manuscript. HJ assisted with acquisition of immunofluorescent data and manuscript preparation. HK and SKoo assisted with protocol development and experimental setup. SKim conceived and designed the study, coordinated the acquisition of all study data, performed analysis and interpretation of results and drafted the manuscript. All co-authors were involved in critical revision of initial drafts.

## Conflict of Interest Statement

The authors declare that the research was conducted in the absence of any commercial or financial relationships that could be construed as a potential conflict of interest.
